# Limited Reliability of the Molecular Detection of *Plasmodium* spp. from Incubated Blood Culture Samples for Forensic Purposes

**DOI:** 10.3390/microorganisms10020406

**Published:** 2022-02-10

**Authors:** Felix Weinreich, Ralf Matthias Hagen, Wibke Loag, Oumou Maïga-Ascofaré, Denise Dekker, Hagen Frickmann, Ulrike Loderstädt

**Affiliations:** 1 Department of Microbiology and Hospital Hygiene, Bundeswehr Hospital Hamburg, 20359 Hamburg, Germany; Felixweinreich@bundeswehr.org; 2 Department of Microbiology and Hospital Hygiene, Bundeswehr Central Hospital Koblenz, 56070 Koblenz, Germany; ralfmatthiashagen@bundeswehr.org; 3 Bernhard Nocht Institute for Tropical Medicine Hamburg, 20259 Hamburg, Germany; loag@bnitm.de (W.L.); maiga@bnitm.de (O.M.-A.); dekker@bnitm.de (D.D.); 4 Kumasi Centre for Collaborative Research in Tropical Medicine, College of Health Sciences, KNUST, Kumasi 039-5028, Ghana; 5 German Center for Infection Research (DZIF), Partner Site Hamburg-Lübeck-Borstel-Riems, 20359 Hamburg, Germany; 6 Institute for Medical Microbiology, Virology and Hygiene, University Medicine Rostock, 18057 Rostock, Germany; 7 Department of Hospital Hygiene & Infectious Diseases, University Medicine Göttingen, 37075 Göttingen, Germany

**Keywords:** *Plasmodium*, routine microscopy, molecular diagnosis, blood culture, real-time PCR, test accuracy, test comparison

## Abstract

The suitability of incubated blood culture material for forensic molecular malaria diagnosis was assessed for non-endemic settings for cases in which the differential diagnosis malaria was initially overlooked. For the proof-of-principle assessment, residual blood culture materials from febrile patients from tropical Ghana were investigated by real-time PCR and compared with available historic microscopic results. In 2114 samples, for which microscopical results and real-time PCR results were available, microscopical results comprised 711 *P. falciparum* detections, 7 *P. malariae* detections, 1 microscopically not-further-discriminable *Plasmodium* spp. detection as well as 13 detections of mixed infections comprising 12 cases of *P. falciparum*/*P. malariae* co-infections and 1 case of a *P. falciparum*/*P. ovale* complex co-infection, while real-PCR indicated 558 *P. falciparum* detections, 95 *P. malariae* detections, 10 *P. ovale* complex detections, 1 *P. vivax* detection and 4 detected *P. falciparum*/*P. malariae* co-infections. Concordance of routine microscopy and real-time PCR was imperfect. Using routine microscopy as reference was associated with a seemingly low agreement of positive real-time PCR results of 90.9%. However, if positive samples, either by routine microscopy or real-time PCR or both, were applied as a combined reference, the agreement of positive results obtained with real-time PCR was increased from 74.0% to 77.9%, while the agreement of positive results obtained with routine microscopy was decreased from 100% to 85.3%. The predictive value of routine microscopy for negative results in the reference was slightly better with 90.9% compared to real-time PCR with 86.9%; the concordance between routine microscopy and real-time PCR was imperfect. In conclusion, even suboptimal sample materials such as incubated blood culture materials can be applied for forensic malaria diagnosis, if more suitable sample materials are not available, but the molecular detection rate of positive results in routine microscopy is much lower than previously reported for non-incubated blood.

## 1. Introduction

Training options to maintain skills in routine malaria microscopy in non-endemic settings are limited because of scarcely available sample material. Morphologically similar species such as *Plasmodium vivax*, *P. ovale curtisi* and *P. ovale wallikeri* tend to be misinterpreted under the microscope [1,2,3] with more than 10% misidentification of *P. vivax* and *P. ovale* complex [1]. *P. ovale curtisi* and *P. ovale wallikeri*, in particular, are virtually indistinguishable by routine microscopy. Molecular approaches for the screening of malaria are useful alternatives for resource-rich settings with limited skills of the laboratory personnel in routine malaria microscopy, as molecular approaches such as real-time PCR are easy to standardize and, thus, less investigator-dependent than routine microscopy. Due to the molecular assays’ high sensitivity [4,5,6], nucleic acid amplification tests for malaria are also useful if a correct identification of early infections with low parasitaemia is desired or mixed plasmodial infections need to be excluded [7,8]. Further, molecular approaches such as PCR are more sensitive than antigen testing, including the recently introduced “ultrasensitive rapid diagnostic tests” [9,10]. Apart from the diagnostic needs in resource-rich non-endemic settings, the suitability of highly sensitive real-time PCR assays has also been suggested for malaria eradication programs [11,12,13,14,15,16].

Whole blood and—even more—capillary blood [17] are the optimum materials for the molecular diagnosis of malaria. In contrast, diagnostic attempts with alternative, less invasively collectible specimens were rather disappointing [18]. Of note, more easily storable and transportable sample materials such as dried whole blood spots are less reliable than whole blood, particularly for the diagnosis of low-density *P. vivax* infections [19]. Alternative sample materials, from which the detection of *Plasmodium* spp. is basically possible if sensitivity loss is accepted, comprise rapid diagnostic tests [20], scrapes of the blood smears on microscopic slides [21] and even stool samples [22]. In spite of sensitivity limitations, there are circumstances under which the use of sub-optimal sample materials needs to be considered for forensic diagnostics. Therefore, it is useful to assess the diagnostic accuracy of real-time PCR-based malaria diagnosis for such instances. In non-endemic settings, a malaria screening is not a routine procedure if malaria is not suspected in case of symptoms of septic fever, especially if a patient does not mention traveling to areas of malaria endemicity. To identify invasive bacterial infections, blood cultures need to be performed. If a blood culture stays negative 5–7 days after the initiation of incubation and the forensic question of an overlooked malaria arises, it needs to be confirmed as to whether real-time PCR from such a material can reliably detect the abundance or absence of microscopically detectable parasites at the time of sample acquisition. If the answer is yes, it can be forensically decided whether malaria would have been microscopically diagnosed at the time of sample acquisition if this assessment had not been initially overlooked.

Unfortunately, molecular diagnostics from incubated blood culture material are not routinely performed. Standard nucleic acid extraction procedures on this type of sample material are associated with inhibition of the PCR reaction, requiring more laborious sample preparation [23]. Nonetheless, molecular malaria diagnostics from incubated blood culture materials still have to be considered in case of a forensic need. The present study aimed at evaluating the diagnostic reliability of real-time PCR-based molecular malaria diagnoses from incubated blood culture materials as compared to traditional routine microscopy from whole-blood samples.

## 2. Materials and Methods

### 2.1. Study Type

The study was designed as a head-to-head test comparison of malaria microscopy and malaria real-time PCR using historic residual sample materials from previous epidemiological studies in Ghana, which had been conducted between 2007 and 2008 [24,25,26,27,28].

### 2.2. Residual Sample Materials Applied for the Malaria Real-Time PCR Testing in This Study

Incubated blood culture material had been collected from a total of 2324 BD (Becton Dickinson, Heidelberg, Germany) blood culture bottles (BACTEC Plus Aerobic/F Medium for adults and BD BACTEC Peds Plus Medium for children) that had been inoculated with blood from febrile Ghanaian patients in the course of a two-year study period from 2007 to 2008 [24,25,26,27,28]. In short, the bottles were filled according to the manufacturer’s instructions and incubated at 36 °C until a positive result was indicated or otherwise for a total of 5 days as detailed elsewhere [24]. Culture-based growth of bacteria succeeded from 12.7% of the samples, while the inclusion of panbacterial 16S rRNA DNA gene-specific PCR increased the proportion of positive samples to 36.7% [25]. Residual samples of the collected blood culture material had been stored at −80 °C and shipped on dry ice to Germany for molecular assessments.

### 2.3. Microscopic Assessment of Freshly Taken EDTA Blood Samples during the Studies in Ghana

In parallel to the acquisition of those blood culture materials, which were reassessed by malaria real-time PCR over the course of this study, EDTA blood samples from the Ghanaian fever patients had been subjected to routine microscopy of Giemsa-stained [29] thick and thin blood films in order to identify *Plasmodium* spp. directly. If malaria parasites were detected, species identification had been performed and parasitemia had been determined [30] and indicated as parasites per microliter. The applied protocol for the microscopic mass screening for malaria was suggested and detailed elsewhere [30]. In short, routine microscopy analysis was carried out by an experienced microscopist, comprising examination of 200 oil immersion fields of the thick blood smear (0.5 µL of blood). Parasitemia was estimated on the basis of 8000 leukocytes per µL as described elsewhere [30]. Data on the patients’ routine malaria microscopy results had been anonymized for this study.

### 2.4. Sample Preparation Prior to PCR Assessment

Due to the above-mentioned PCR inhibition risk, DNA was extracted by applying a previously described laborious centrifugation-based procedure [24] instead of a commercial standard procedure and stored at −80 °C. The centrifugation-based procedure, which was in fact more a purification of bacteria as well as of free DNA from the blood culture samples for PCR rather than a traditional nucleic acid extraction, was reported in detail elsewhere [24]. In short, erythrocytes of 1 mL blood culture sample were sedimented by centrifugation at 140× *g* for 10 min, before the supernatant of 800 μL was transferred into another tube, while the sediment was discarded. A volume of 400 μL double-distilled water was added to the 800 µL supernatant to ensure the lysis of remaining erythrocytes. Subsequently, bacterial cells and free DNA within the sample were sedimented by centrifugation at 750× *g* for an additional 10 min. After discarding the supernatant, the remaining pellet became resuspended and washed in 1 mL double-distilled water with additional centrifugation at 750× *g* for 10 min. Again, the supernatant was discarded and the pellet was resuspended in 200 μL double-distilled water. In a final step, the sample was incubated for 10 min at 95 °C to release DNA from bacterial cells. The proof-of-principle of the suitability of this centrifugation approach had been performed with real-time PCRs targeting entero-invasive bacteria [24] as well as with pan-bacterial real-time PCRs [25]. In order to ensure the reproducibility of the centrifugation-based DNA purification approach, repeated incubation of blood culture bottles with different concentrations of the same *Salmonella enterica* strain had been performed. After incubation until the lag phase of bacterial growth and centrifugation-based purification of DNA as described above, cycle threshold (Ct) values of *Salmonella* spp.-specific real-time PCR [24] were reproducible in all samples with a standard deviation range of less than 0.5 cycle threshold (Ct) steps both from fresh and from frozen incubated blood culture samples. The effect of freezing, in contrast, resulted in a loss of 2 Ct steps.

### 2.5. Applied Malaria Screening Real-Time PCR Assay

Screening was performed with a SybrGreen-based non-commercial real-time PCR targeting the 18S rRNA gene of *Plasmodium* spp. [31]. The primers of the SybrGreen-based screening assay [31] for malaria are provided in Table 1 for readers with interest in molecular details, and the positive control plasmid inserts of the *Plasmodium* spp. with medical relevance, as applied for the melting temperature definitions, are presented in Appendix A, Table A1. As shown in a recent assessment, the SybrGreen-based screening PCR is associated with a sensitivity of 94.8% and a specificity of 100.0% if applied with whole blood, as observed with a sample collection from patients with microscopic and sub-microscopic malaria [32].

### 2.6. Confirmatory Malaria Real-Time PCR Assay and Conditions under Which This Confirmation Was Performed

For cases of uncertain or contradictory non-commercial SybrGreen-based real-time PCR screening results, differentiation at the species level and screening for infections with more than one *Plasmodium* species were attempted using a limited number of 605 available reactions of the RealStar Malaria S&T PCR Kit 1.0 (Altona Diagnostics, Hamburg, Germany), which included an inhibition control reaction for those 605 samples. The assay was applied as described by the manufacturer’s instructions and as detailed elsewhere [2,32]. This kind of confirmation testing was applied for the following indications in declining order of priority: (1) SybrGreen-based screening PCR reactions with distorted melting curves within the expected temperature range potentially indicating infections with more than one *Plasmodium* species or non-specific reactions, (2) samples with positive routine microscopy but negative SybrGreen-based screening PCR in order to discriminate PCR inhibition events from potentially false-positive microscopic results, and (3) lowest-priority samples with low cycle threshold values (Ct 15–25) and melting curves suggesting *P. falciparum* mono-infection in SybrGreen-based screening PCR, as such situations are conspicuous for “hidden” double infections with highly differing proportions of parasitemia, as shown previously [2]. If SybrGreen-based screening PCR and commercial Altona Diagnostics confirmation PCR were performed due to distorted melting curves in the SybrGreen-based screening PCR and conflicting results of both were seen, the results of the commercial Altona Diagnostics differentiation PCR were accepted.

### 2.7. Differentiation PCR Assay for the Discrimination of P. ovale wallikeri and P. ovale curtisi

In case of detection of the *P. ovale* complex either by routine microscopy, using the non-commercial SybrGreen-based screening assay, or with the commercial Altona Diagnostics differentiation assay, a non-commercial duplex real-time PCR, as described before [33] and as associated with high diagnostic accuracy in comparison to competitor assays [3,34,35], was added for the discrimination of *P. ovale curtisi* and *P. ovale wallikeri*. The oligonucleotides of this latter differentiation assay are given in Table 1, and the positive control plasmids are included in Appendix A, Table A2. The *P. ovale* complex differentiation assay was performed as described previously [3].

**Table 1:** Oligonucleotide sequences of the applied non-commercial malaria real-time PCRs.

Forward Primer Name	Forward Primer Sequence	Reverse Primer Name	Reverse Primer Sequence	Probe Name	Probe Sequence
SybrGreen-based generic *Plasmodium* spp.-specific real-time PCR targeting the 18S rRNA gene used for screening, according to Mangold et al. [32].					
PL1473F18	5′-TAACGAACGAGATCTTAA-3′	L1679R18	5′-GTTCCTCTAAGAAGCTTT-3′	n.a.	n.a.
Dual hybridization probe real-time PCR for the discrimination of *P. ovale curtisi* and *P. ovale wallikeri* targeting the ssu rRNA, according to Bauffe et al. [34]					
POF	5′-ATAAACTATGCCGACTAGGTT-3′	POR	5′-ACTTTGATTTCTCATAAGGTACT-3′	pPOC ^1^	5′-TTCCTTTCGGGGAAATTTCTTAGA-3′
				pPOW ^2^	5′-AATTCCTTTTGGAAATTTCTTAGATTG-3′

n.a. = not applicable. pPOC = probe for the detection of *P. ovale curtisi*. pPOW = probe for the detection of *P. ovale wallikeri*. ^1^ FAM and BHQ1 were used as reporter and quencher, respectively. ^2^ JOE and BHQ1 were used as reporter and quencher, respectively.

### 2.8. Inclusion and Exclusion Criteria

Samples were included in the assessment if at least enough sample material for the SybrGreen-based screening PCR, i.e., a 5 µL volume, was available, leading to the exclusion of three samples. The lack of routine microscopy results was no exclusion criterion for the PCR assessment. Samples with lacking microscopic results were nevertheless included in the molecular prevalence assessment for the sample collection.

### 2.9. Assessment Strategy for the Obtained Data

Considering the well-known sensitivity differences of real-time PCR and routine microscopy [4,5,6,7,8,32], combined references were applied for the agreement and concordance assessment. In a first step, microscopic results were considered as the reference for the calculation of the agreement of positive and negative results, as well as predictive values of PCR for positive and negative results in the reference. Keeping the known reduced sensitivity of routine microscopy compared to PCR in mind [4,5,6,7,8,32], a second calculation was added, which considered a sample to be positive for malaria if at least a positive microscopic result or a positive PCR result were available to calculate agreement of positive results as well as predictive values of PCR and routine microscopy for negative results obtained with this combined reference.

Next to qualitative real-time PCR results, semi-quantitative results based on cycle threshold (Ct) values as well as microscopically quantified parasitemia were recorded and descriptively assessed. The correlation (Spearman r) of Ct values obtained with the SybrGreen-based screening PCR and microscopically observed parasitemia was calculated.

### 2.10. Ethics

For this study, ethical clearance by the medical association of Hamburg, Germany (reference number: WF-011/19; obtained on 11 March 2019) allowed the anonymous use of residual sample volumes, which were labeled with laboratory-derived sample-ids, without further requirements such as informed consent. Anonymization was performed in line with national German laws, i.e., the data protection regulation (“Datenschutzgrundverordnung”). Since the data and sample materials had been anonymized, no patient-specific details can be connected to the results presented here, which is an admitted deviation from the STARD (Standards for Reporting Diagnostic Accuracy) criteria [36].

## 3. Results

### 3.1. Overall Assessment

From the 2321 samples included in the analysis, results of the microscopic analysis were available for 2114 samples. Within the 207 samples without microscopic results, real-time PCR identified 58 samples containing *Plasmodium* spp. DNA, comprising 44× *P. falciparum*, 12× *P. malariae*, 1× *P. vivax* and 1× *P. ovale complex*. Those 207 samples were excluded from the test comparison but all PCR-based detections are indicated in Appendix A, Table A3. A flowchart indicating the exclusions is provided in Table 2.

In a total of 732 out of the 2114 included cases, malaria was microscopically diagnosed with 711 *P. falciparum* detections, 7 *P. malariae* detections, 1 microscopically not-further-discriminable *Plasmodium* spp. detection and 13 detections of mixed infections comprising 12 cases of *P. falciparum*/*P. malariae* co-infections and 1 case of a *P. falciparum*/*P. ovale* complex co-infection.

In a total of 211 microscopically positive cases, SybrGreen PCR-based screening was negative and, thus, commercial hybridization probe-based Altona Diagnostics confirmation PCR was added to assess the relevance of sample inhibition within these negatively tested samples. Relevant PCR inhibition was not recorded, but plasmodial DNA was recorded only in a minority of 69 out of 211 samples by confirmation testing with the Altona Diagnostics assay as well.

Out of 740 initially positive SybrGreen-screening PCR results, 229 showed distorted melting curves in the expected temperature range of *Plasmodium*-specific amplicons, so commercial Altona Diagnostics real-time multiplex PCR was applied as a specificity control. From those 229 cases, 14 SybrGreen-based screening PCR signals were excluded as non-specific due to lacking confirmation by commercial hybridization probe-based Altona Diagnostics real-time PCR. In total, four cases of mixed infections could be confirmed using the commercial molecular approach by Altona Diagnostics among the samples with distorted melting curves in the SybrGreen-based screening PCR, each comprising combinations of *P. falciparum* and *P. malariae*.

Finally, exclusion of infections due to different *Plasmodium* spp. in samples with low cycle threshold (Ct) values (assessed range from Ct 15 to Ct 25) in SybrGreen-based screening PCR and melting curves indicating *P. falciparum* by applying commercial Altona Diagnostics multiplex real-time PCR succeed in 165 instances. Multi-infections were not recorded. Of note, 12 samples with melting curves unambiguously indicating *P. falciparum* in the SybrGreen-based screening assay were missed by the commercial hybridization probe-based Altona Diagnostics real-time PCR approach.

Considering the abovementioned corrections for specificity to control uncertain results of the SybrGreen-based screening PCR, which are detailed in Table 3, real-time PCR resulted in 558 *P. falciparum* detections, 95 *P. malariae* detections, 10 *P. ovale* complex detections, 1 *P. vivax* detection and 4 detected *P. falciparum*/*P. malariae* co-infections within the 2114 included samples. Imperfect concordance of PCR and routine microscopy was also recorded at the species level as shown in Table 4 and in Appendix A, Table A3.

Focusing on quantitative parameters, microscopically diagnosed parasitemia ranged from the detection limit (<50 parasite/µL) to 1,859,200 parasites/µL, with a mean of 108,771 parasites per µL (±standard deviation SD 188,491 parasites per µL) and a median of 29,917 parasites per µL in a left-shifted distribution. Recorded Ct-values of the SybrGreen-based screening PCR ranged from 15 to 37 (mean ± SD: 27.71 ± 4.10; median: 28).

All 11 *P. ovale* complex infections as identified within the 2321 samples were subjected to real-time PCR-based discrimination of *P. ovale curtisi* and *P. ovale wallikeri*. Thereby, *P. ovale curtisi* was identified in two instances, and *P. ovale wallikeri* was identified in six instances, while discrimination failed in three instances.

### 3.2. Agreement of Real-Time PCR and Routine Microscopy

Agreement of real-time PCR and routine microscopy results was calculated both for routine microscopy as the reference and for a combined reference comprising routine microscopy and real-time PCR. As indicated in Table 5, both the agreement of positive results and the predictive value of real-time PCR for negative results in the reference were lower than recorded for routine microscopy if a combined reference comprising both positive real-time PCR results and routine microscopy results was used. If routine microscopy was applied as the reference, the agreement of positive results of real-time PCR was even lower, and the agreement of negative results and predictive value for positive results in the reference were low as well.

### 3.3. Analysis of the Distribution of Ct-Values and Parasitemia

SybrGreen-based screening PCR Ct-values of *Plasmodium* spp. detections with concordant and discordant detections in routine microscopy were compared and the results are outlined in Table 6. Ct values of the commercial Altona Diagnostics PCR were not included as not all samples were assessed applying this diagnostic approach. Mixed infections were excluded from this analysis. Slightly higher average Ct values in PCR-positive but routine microscopy negative samples suggested a higher baseline parasitemia as the limit of detection for routine microscopy compared to PCR. However, overlapping standard deviation (SD) ranges indicated only a minor effect. Focusing on parasitemia, lower average parasitemia of *P. falciparum* but higher average parasitemia of *P. malariae* were recorded in routine microscopy-positive, PCR-negative samples compared to samples that were positive in both diagnostic approaches (Table 7). Correlation of the Ct values of the SybrGreen-based screening PCR and microscopically observed parasitemia resulted in a Spearman r value of −0.42 with a significance level of *p* < 0.0001.

## 4. Discussion

The study was performed to assess the suitability and reliability of real-time PCR from incubated blood culture material compared to traditional routine microscopy by re-assessing historical samples from a previous epidemiological study [24,25,26,27,28]. As expected for a highly sensitive approach such as real-time PCR [4,5,6], its addition to traditional routine microscopy resulted in a considerable increase in malaria-positive samples compared to the microscopical approach alone. Not surprisingly, if routine microscopy was applied as a reference, real-time PCR was associated with a low agreement of negative results and a sub-optimal predictive value for positive results in the reference. However, if positive samples, either by routine microscopy or real-time PCR or both, were applied as a composite reference, agreement of positive results obtained with real-time PCR was increased while agreement of positive results obtained with routine microscopy was decreased. The predictive value of routine microscopy for negative results in the references was slight compared to real-time PCR and the overall concordance between routine microscopy and real-time PCR was rather low. Regarding the cycle threshold (Ct) values and the microscopically observed parasitemia, only a weak-to-moderate negative correlation was recorded, a result that is well in line with previous observations [2].

The observed discordance regarding the positive results in real-time PCR and routine microscopy requires further interpretation. Real-time PCR on EDTA blood is more sensitive than routine microscopy [2,32], which could explain the samples additionally detected by real-time PCR but not by routine microscopy in our study. It must be considered, however, that the samples used were diluted in blood culture medium, whereby DNA might have been degraded during the incubation step and, further, intracellular parasite DNA could have been lost during the pre-analytic centrifugation steps. Such DNA loss was not observed in a previous real-time PCR screening for invasive enteropathogenic and other bacteria [24,25] with the same samples, in which even higher sensitivity of real-time PCR was seen compared to bacterial growth on agar plates. However, in contrast to bacterial DNA, plasmodial DNA is not enriched in blood culture and intracellular parasites may easily become lost during the centrifugation step. These combined effects potentially reduced the agreement of positive real-time PCR from blood culture material with the applied references compared to real-time PCR from EDTA blood or fresh capillary blood, respectively. According to previous studies [17,37], capillary blood buffy coat samples proved to be particularly reliable regarding the differentiation and quantification of plasmodia [37], while differences between capillary and venous blood films are still controversial [17,37] and the time of sampling under therapy is also known to affect the recorded parasitemia [38]. Conclusions on differences in the reliability of molecular malaria detection with incubated blood culture materials and other suboptimal sample materials such as dried blood spots [19], rapid diagnostic tests [20], microscopical slides [21] or stool [22] are, unfortunately, difficult to draw, because the applied study designs varied considerably. Of note, malaria-positive samples were recognized by real-time PCR at the detection limit of 50 parasites/µL and in the case of submicroscopic malaria as well in our study. This finding speaks in favor of the alternative hypothesis that factors other than just parasitemia could have affected the real-time PCR’s reliability from the assessed blood culture media, leading to stochastic PCR failure rather than a reduced limit of detection. The slightly higher cycle threshold values in PCR-positive routine microscopy-negative samples point in this direction as well.

Positive samples that were identified by routine microscopy but came up negative by PCR are much more difficult to explain than vice versa. Firstly, inhibition-control PCR, as included in the commercial Altona Diagnostics real-time PCR approach, did not indicate relevant inhibition within the assessed routine microscopy-positive but PCR-negative samples. Thus, PCR inhibition did not play a relevant role in these reactions. In line with this, commercial Altona Diagnostics real-time PCR mostly reproduced the results of SybrGreen-based screening PCR in case of conclusive melting curves. There was only a minority of 14 samples that were unambiguously positive by SybrGreen-based screening PCR but missed by the hybridization probe-based confirmatory Altona Diagnostics real-time PCR. This discrepancy can be explained by a slight discordance in diagnostic accuracy of the applied PCR assays, as reported recently [32]. Secondly, the comparison of the parasitemia observed microscopically in PCR-positive and PCR-negative samples did not exhibit a relevantly lower parasitemia in samples missed by PCR. These two arguments raise the question as to whether false-positive routine malaria microscopy results may at least have partially contributed to the proportion of routine microscopy-positive but PCR-negative samples. Unfortunately, this question is difficult to answer in the absence of a “true gold standard” that has a perfect diagnostic accuracy [39]. However, the described information speaks in favor of a considerable proportion of false-positive samples among the routine microscopy-positive but PCR-negative samples. If this assumption was true, the “true” agreement of positive real-time PCR from blood culture material would be even higher than recorded in this study. Hypothetically, high concentrations of bacterial DNA from bacteria grown in the blood samples might also have interacted with the malaria PCR reactions. However, correlations of bacterial detections in the blood cultures and malaria PCR results were not attempted in this study. As stated above in the “Materials and Methods” chapter, bacterial culture and pan-bacterial PCR with subsequent Sanger sequencing provided considerably different positivity rates for the assessed blood culture materials [24,25]. Similar cycle threshold (Ct) values made the discrimination of missed bacterial growth due to inappropriate pre-analytic conditions in the tropics and secondary contamination of the samples unreliable. Due to the resulting uncertainty, the positivity of blood culture material for bacterial pathogens was not included as a parameter in the anonymous data collection for this study. Of note, cross-reaction of the malaria PCRs with bacterial DNA was not excluded, although the eukaryotic DNA sequences serving as targets for the malaria PCRs, which do not occur in prokaryotic cells, do not make this option very likely.

Of note, there was only a minor difference between the recorded cycle threshold (Ct) values of samples, which were concordantly positive by routine microscopy and PCR as compared to the PCR-positive routine microscopy-negative samples. This finding confirms a previous report on a weak association between microscopically observed parasitemia in EDTA blood and recorded Ct-values [2], which was the same as the recorded weak-to-moderate correlation in this study, as stated above.

Of further interest, SybrGreen-based screening PCR from blood culture specimens resulted in a high proportion of distorted melting curves, a phenomenon that is virtually absent in EDTA blood samples in our experience [31,32]. This required hybridization probe-based confirmatory testing for a discrimination at the species level, involving the application of the Altona Diagnostics assay. Thereby, a minority of 14 samples could not be confirmed as malaria positive. One reason could be that those 14 SybrGreen-based screening PCR signals were indeed non-specific, as decided for the interpretation algorithm. An alternative hypothesis is that the hybridization probe-based Altona Diagnostics confirmatory approach might have missed some “true positive” samples itself. This is not unlikely due to the slight discordance in diagnostic accuracy of the applied PCR assays, as reported recently [32]. However, because the study design was based on the SybrGreen PCR-based screening approach and the hybridization probe-based Altona Diagnostics confirmatory approach was added only in the case of questionable SybrGreen-based screening PCR results, this question cannot be answered. It remains unclear as to how many samples would have been finally missed by the Altona Diagnostics confirmation PCR if it had been used as the screening approach. This is an admitted limitation of the study, as detailed below.

As a side finding of the study, sympatric co-occurrence of *P. ovale wallikeri* and *P. ovale curtisi* could be confirmed for Ghana; a finding matching the results obtained from travel returnees from Western Africa [3]. Of note, a minority of samples did not contain sufficient target DNA to allow a conclusive discrimination. Further, the occurrence of *P. vivax* in Ghana is considered as not confirmed so far [40]. In this study, two real-time PCR signals that were suggestive of the abundance of low amounts of *P. vivax* DNA, but no microscopical proof, were recorded. One of the two real-time PCR signals occurred in a sample completely without microscopic assessment. These findings are indicative of either submicroscopic *P. vivax* occurrence at very low frequency in Ghana or solitary non-specific real-time PCR results as an alternative hypothesis.

The study has a number of limitations. First, the applied residual sample materials were old, potentially reducing the diagnostic reliability. However, appropriate storage at −80 °C was assured, making severe sample age-associated DNA degradation unlikely. Most likely still adequate sample quality was also suggested by the fact that the inhibition control assay of the commercial Altona Diagnostics PCR kit did not indicate any relevant sample inhibition and, thus, degradation products potentially interfering with nucleic acid amplification did not play a major role. This finding further supports our decision on working with historic residual sample materials. As a second limitation, the confirmatory commercial Altona Diagnostics real-time PCR assay, including the inhibition control PCR reaction, could not be performed for all samples for economic reasons. Third, the study did not contain a reference with perfect diagnostic accuracy, so only comparative evaluations could be performed.

## 5. Conclusions

The study was conducted to answer the question of whether real-time PCR from incubated blood culture material can reliably identify the presence or absence of malaria parasitemia at the time of the sample acquisition for forensic purposes. In spite of the limitations resulting from the use of historic samples, it has to be concluded that real-time PCR from such specimens is not accurate enough. Accordingly, a diagnostic attempt with incubated blood culture material is only justified in cases in which initially missed malaria infections shall be confirmed for the time point of blood culture acquisition due to forensic reasons and more suitable samples are unavailable. Such PCR results have to be interpreted with care since the results of real-time PCR from blood culture material are considerably less reliable than from EDTA blood [2,32] or fresh capillary blood [17]. Compared to easy-to-collect, more reliable samples such as EDTA blood [2,32], incubated blood culture materials are also poor specimens for epidemiological assessments requiring diagnostic accuracy-adjusted prevalence estimations [41].

## Figures and Tables

**Table 2:** Flow-chart indicating the diagnostic workflow with residual materials from incubated blood culture samples from a previous Ghanaian study [24,25,26,27,28] as detailed above in the “Materials and Methods” chapter.

2321 Blood Culture Samples from a Ghanaian Epidemiological Study Included, of Which 2114 Had Microscopical Results from Concomitantly Taken Whole-Blood Samples
↓
Assessment of all 2321 blood culture sample residual volumes by SybrGreen-based non-commercial malaria real-time PCR screening
↓
Inclusion of 605/2321 (26.1%) blood culture sample residual volumes (comprising 122 samples without available microscopic result) into confirmatory commercial Altona Diagnostics real-time PCR testing, if the following conditions were met: Samples were microscopically positive but negative in SybrGreen-based screening PCR (*n* = 211); Distorted melting curves of the SybrGreen-based screening PCR within the expected temperature range did not allow unambiguous interpretation (*n* = 229); *Plasmodium falciparum* mono-infections were detected by SybrGreen-based screening PCR with low cycle threshold values (15–25) and, thus, mixed plasmodial infections with severely dysbalanced parasitemia could not be excluded by melting curve analysis alone (*n* = 165).
↓
Non-commercial duplex real-time PCR for the discrimination of *P. ovale wallikeri* and *P. ovale curtisi* in case of *P. ovale* complex detections by real-time PCR or by routine microscopy (*n* = 11)

↓ Direction of the diagnostic workflow.

**Table 3:** Applied interpretation algorithm for the confirmatory testing applying the commercial hybridization probe-based Altona Diagnostics real-time PCR assay (*n* = 605).

SybrGreen PCR-Based Screening Result	Result of Hybridization Probe-Based Confirmatory Testing Applying the Altona Diagnostics Assay	Diagnostic Interpretation Assumed as “True Result” Considering Both PCR Reactions	Number (*n*)
*P. falciparum*	*P. falciparum*	*P. falciparum*	153
	Negative	*P. falciparum* *	12
Negative	*P. falciparum*	*P. falciparum*	63
	*P. malariae*	*P. malariae*	3
	*P. ovale* complex	*P. ovale* complex	3
	Negative	Negative	142
Distorted melting curves within the expected temperature range	*P. falciparum*	*P. falciparum*	210
	*P. falciparum*/ *P. malariae* co-infection	*P. falciparum*/*P. malariae* co-infection	4
	*P. malariae*	*P. malariae*	1
	Negative	Negative	14

* The interpretation was due to unambiguous melting curves in the SybrGreen-based screening PCR, strongly suggesting incorrect results in the hybridization probe-based assay.

**Table 4:** Distribution of *Plasmodium* species either by routine microscopy or PCR-based detection in the incubated blood culture materials. Only samples are shown that had been assessed by both microscopy and PCR (*n* = 2114).

Kind of Infection	Detected by Routine Microscopy, *n*/*n* (%)	Detected by Real-Time PCR, *n*/*n* (%)	Concordance of Routine Microscopy and PCR, *n*/*n* (%)	Detected in Total Combining Routine Microscopy and Real-Time PCR, *n*/*n*, %
*Plasmodium falciparum*	711/2114 (33.6%)	558/2114 (26.4%)	1773/2114 (83.9%)	806/2114 (38.1%)
*Plasmodium vivax*	0/2114 (0.0%)	1/2114 (0.1%)	2113/2114 (99.9%)	1/2114 (0.1%)
*Plasmodium ovale* complex	0/2114 (0.0%)	10/2114 (0.5%)	2104/2114 (99.5%)	10/2114 (0.5%)
*Plasmodium malariae*	7/2114 (0.3%)	95/2114 (4.5%)	2018/2114 (95.5%)	99/2114 (4.7%)
*Plasmodium* spp. (not further discriminated)	1/2114 (0.1%)	0/2114 (0.0%)	2113/2114 (99.9%)	1/2114 (0.1%)
Infection with more than one *Plasmodium* spp.	13/2114 (0.6%) ^1^	4/2114 (0.2%) ^2^	2097/2114 (99.20%)	17/2114 (0.8%)

^1^ Details of mixed infections as detected by routine microscopy: *P. malariae* and *P. falciparum* (*n* = 12), *P. falciparum* and *P. ovale* complex (*n* = 1). ^2^ Details of mixed infections as detected by real-time PCR: *P. malariae* and *P. falciparum* (*n* = 4).

**Table 5:** Agreement of positive and negative real-time PCR results with routine microscopy as the reference and with a combined reference comprising routine microscopy and real-time PCR regarding malaria detection at the genus level (*n* = 2114).

Criterium	Real-Time PCR (with Routine Microscopy as the Reference)	Real-time PCR (with a Combined Reference of Routine Microscopy and Real-Time PCR)	Routine Microscopy (with a Combined Reference of Routine Microscopy and Real-Time PCR)
Agreement of positive results, *n*/*n* (%)	542/732 (74.0%)	668/858 (77.9%)	732/858 (85.3%)
Agreement of negative results, *n*/*n* (%)	1256/1382 (90.9%)	n.a.	n.a.
Predictive value for positive results in the chosen reference, *n*/*n* (%)	542/668 (81.1%)	n.a.	n.a.
Predictive value for negative results in the reference, *n*/*n* (%)	1256/1446 (86.9%)	1256/1446 (86.9%)	1256/1382 (90.9%)

n.a. = not applicable.

**Table 6:** SybrGreen-based screening PCR Ct values of *Plasmodium* spp. detections for samples that were positive in both routine microscopy and real-time PCR and in real-time PCR only (*n* = 2114).

Species	Recorded Ct-Values in Case of Concordance of Routine Microscopy and PCR, *n*; Mean (± SD); Median (Minimum, Maximum)	Recorded Ct-Values with Diagnosis Based on PCR only, *n*; Mean (±SD); Median (Minimum, Maximum)
*P. falciparum*	*n* = 465; 26,4 (±3.9); 26.5 (15.4, 37.0)	*n* = 562; 26.8 (±3.9); 27.0 (15.0, 37.0)
*P. vivax*	*n* = 0; n.a.	*n* = 1; 32.4 (±0); 32.4 (32.4, 32.4)
*P. ovale* complex	*n* = 0; n.a.	*n* = 10; 33.0 (±2.2); 33.2 (29.0, 35.7)
*P. malariae*	*n* = 3; 30.6 (±1.5); 31.5 (28.5, 31.7)	*n* = 99; 31.7 (±2.1); 31.7 (24.8, 36.3)

n.a. = not applicable.

**Table 7:** Microscopically diagnosed parasitemia in parasites/µL as recorded for *Plasmodium* spp. detections in both routine microscopy and real-time PCR and in routine microscopy only.

Species	Recorded Parasitemia in Case of Concordance of Routine Microscopy and PCR, *n*; Mean (±SD); Median (Minimum, Maximum)	Recorded Parasitemia with Diagnosis Based on Routine Microscopy only, *n*; Mean (±SD); Median (Minimum, Maximum)
*P. falciparum*	*n* = 463; 142,554.1 (±213,046.9); 63,800 (71, 1,859,200)	*n* = 722; 109,950.5 (±189,177.7); 31,304 (70, 1,859,200)
*P. vivax*	*n* = 0; n.a.	*n* = 0; n.a.
*P. ovale* complex	*n* = 0; n.a.	*n* = 0; n.a.
*P. malariae*	*n* = 3; 665.3 (±645.4); 384 (54, 1558)	*n* = 20; 36,100.3 (±70,620.7); 3079.5 (54, 272,160)

n.a. = not applicable.

## Data Availability

All relevant data are provided within the manuscript. Raw data can be provided on reasonable request.

## Appendix A

**Table A1:** Sequence inserts for the positive control plasmids of the SybrGreen-based malaria screening PCR included in a pEX-A128 vector backbone as applied for the definition of the melting temperatures.

Positive Control Insert Based on the *P. falciparum* Sequence according to the NCBI GenBank Accession Number JQ627151.1.
5′-TTCCGATAACGAACGAGATCTTAACCTGCTAATTAGCGGCGAGTACACTATATTCTTATTTGAAATTGAACATAGGTAACTATACATTTATTCAGTAATCAAATTAGGATATTTTTATTAAAATATCCTTTTCCCTGTTCTACTAATAATTTGTTTTTTACTCTATTTCTCTCTTCTTTTAAGAATGTACTTGCTTGATTGAAAAGCTTCTTAGAGGAACATTGTG-3′
Positive Control Insert Based on the *P. malariae* Sequence According to the NCBI GenBank Accession Number EF467831.1.
5′-TTCCGATAACGAACGAGATCTTAACCTGCTAATTAGCGGTAAATACACTATATTCTTAAGTGAAATTAGAATATAGATAAATTGTGCTAATTTTGATTAAAATATTAGAATGTTTTTTTTAATAAAAACGTTCTTTTCCCTTTTTTTCTTAATTATGCATATTTATTTTTTTTCTTCTTTTGCATAAGAATGTATTTGCTTAATTGTAAAGCTTCTTAGAGGAACGATGTG-3′
Positive Control Insert Based on the *P. vivax* Sequence According to the NCBI GenBank Accession Number JQ627158.1.
5′-TTCCGATAACGAACGAGATCTTAACCTGCTAATTAGCGGCAAATACGATATATTCTTACGTGGGACTGAATTCGGTTGATTTGCTTACTTCGAAGAAAATATTGGGATACGTAACAGTTTCCCTTTCCCTTTTCTACTTAGTTCGCTTTTCATACTGTTTCTTTTTCGCGTAAGAATGTATTTGCTTGATTGTAAAGCTTCTTAGAGGAACGATGTG-3′
Positive Control Insert Based on the *P. ovale* complex Sequence According to the NCBI GenBank Accession Number KF018659.1.
5′-TTCCGATAACGAACGAGATCTTAACCTGCTAATTAGCGGCGAATACGTTATATTCCTACTTGAAATTGAATATAGCTGAATTTGCTTATTTTGAAGAATATATTAGGATACATTATAGTGTCCTTTTCCCTTTTCTACTTAATTCGCTATTCATGCTGTTTCTTTTTTGTGTAGGAATGTATTCGTTTGATTGTAAAGCTTCTTAGAGGAACGATGTG-3′
Positive Control Insert Based on the *P. knowlesi* Sequence According to the NCBI GenBank Accession Number KJ917904.1.
5′-TTCCGATAACGAACGAGATCTTAACCTGCTAATTAGCGGCAAATACGATATATTCTTATGTAGAATTGAATATAGTGGATTTGTTAGATTTTGAAGAAAATATTGGAATTACGTTAAATGTGATTCCTTTCCCTTTTCTACTTAATTTACATTTCCATCTATTTCTTTTTTGCGTATGAATGTATTTGCTTGATTGTAAAGCTTCTTAGAGGAACGATGTG-3′

**Table A2:** Sequence inserts for the positive control plasmids of the differentiation PCR for the discrimination of *P. ovale curtisi* and *P. ovale wallikeri* included in a pEX-A128 vector backbone.

Positive Control Insert Based on the *P. ovale curtisi* Sequence according to the NCBI GenBank Accession Number KX672023.1.
5′-AATCTTAACCATAAACTATGCCGACTAGGTTTTGGATGAAACATTTTTAAATAAGAAAATTCCTTTCGGGGAAATTTCTTAGATTGCTTCTTTCAGTACCTTATGAGAAATCAAAGTCTTTGGGTTC-3′
Positive Control Insert Based on the *P. ovale wallikeri* Sequence According to the NCBI GenBank Accession Number MG241227.1.
5′-AATCTTAACCATAAACTATGCCGACTAGGTTTTGGATGAAAGATTTTTAAATAAGAAAATTCCTTTTGGAAATTTCTTAGATTGCTTCCTTCAGTACCTTATGAGAAATCAAAGTCTTTGGGTTC-3′

**Table A3:** Distribution of *Plasmodium* species by routine microscopy or PCR-based detection in the incubated blood culture materials (*n* = 2321).

	Routine Microscopy	*Plasmodium falciparum, n*, Mean Ct (±SD), Parasitemia (±SD)	*Plasmodium vivax, n*	*Plasmodium ovale* Complex, *n*	*Plasmodium malariae, n*, Mean Ct (±SD), Parasitemia (±SD)	*Plasmodium* spp. (not Further Discriminated), *n*, Mean Ct (±SD), Parasitemia (±SD)	Not Performed, *n*, Mean Ct (±SD)	Negative, *n*, Mean Ct (±SD)
Real-Time PCR								
*Plasmodium falciparum*		465; 26.42 (±3.9); 142,554.06 (±213,046.92)				5; 25.34 (±2.04), 118,951.20 (± 96,214.95)	44; 27.27 (±3.87)	92; 28.64 (±3.29)
*Plasmodium vivax*			0			0	1; 35.66 (±0)	1; 32.35 (±0)
*Plasmodium ovale* complex				0		2; 32.27 (±3.27), 8322.50 (±4573.50)	1; 30.00 (±0)	8; 33.13 (±1.86)
*Plasmodium malariae*					3; 30.55 (±1.46); 665.33 (±645.43)	2; 28.83 (±0.91), 2168.50 (±1350.50)	12; 32.47 (±1.77)	94; 31.78 (±2.10)
Negative		248; 0 (±0); 51,460 (±116,560.22)	0	0	4; 0 (±0); 23,964.50 (±39,550.28)	5; 0 (±0); 2103.25 (±1482.74)	149	1256
